# Hitchhiker's Guide to Voltammetry: Acute and Chronic Electrodes for in Vivo Fast-Scan Cyclic Voltammetry

**DOI:** 10.1021/acschemneuro.6b00393

**Published:** 2017-02-09

**Authors:** Nathan T. Rodeberg, Stefan G. Sandberg, Justin A Johnson, Paul E. M. Phillips, R. Mark Wightman

**Affiliations:** † Department of Chemistry, University of North Carolina at Chapel Hill, Chapel Hill, North Carolina 27599-3290, United States; ‡ Neuroscience Center, University of North Carolina at Chapel Hill, Chapel Hill, North Carolina 27599-3290, United States; § Department of Psychiatry and Behavioral Sciences, University of Washington, Seattle, Washington 98195-6560, United States; ‖ Department of Pharmacology, University of Washington, Seattle, Washington 98195-6560, United States

**Keywords:** Fast-scan cyclic voltammetry, carbon-fiber microelectrodes, dopamine, principal component regression, chemometrics

## Abstract

Fast-scan cyclic voltammetry (FSCV) has been used for over 20 years to study rapid neurotransmission in awake and behaving animals. These experiments were first carried out with carbon-fiber microelectrodes (CFMs) encased in borosilicate glass, which can be inserted into the brain through micromanipulators and guide cannulas. More recently, chronically implantable CFMs constructed with small diameter fused-silica have been introduced. These electrodes can be affixed in the brain with minimal tissue response, which permits longitudinal measurements of neurotransmission in single recording locations during behavior. Both electrode designs have been used to make novel discoveries in the fields of neurobiology, behavioral neuroscience, and psychopharmacology. The purpose of this Review is to address important considerations for the use of FSCV to study neurotransmitters in awake and behaving animals, with a focus on measurements of striatal dopamine. Common issues concerning experimental design, data collection, and calibration are addressed. When necessary, differences between the two methodologies (acute vs chronic recordings) are discussed. The topics raised in this Review are particularly important as the field moves beyond dopamine toward new neurochemicals and brain regions.

## Graphical abstract

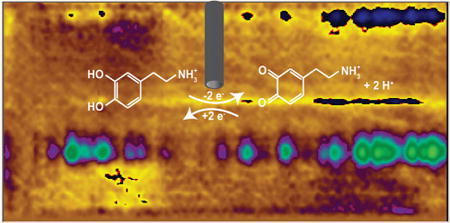

## Techniques for Monitoring Molecules in Neuroscience

The monitoring of molecules in the brain has undergone significant advances in the past four decades. One of the earliest techniques for measuring neurotransmitter release was push–pull perfusion, a method that uses a cannula for sample collection prior to downstream analysis.^1,2^ However, the direct interface of the perfusate with brain tissue raised concerns with sample contamination and flow-induced damage to the surrounding environment. To address these issues, this procedure was later adapted to incorporate a dialysis membrane, creating the technique known as microdialysis.^3–6^ Microdialysis restricts flow to the probe, which minimizes brain damage and maintains sample purity. Equilibration of analytes across the membrane according to their concentration gradients results in concentration changes in the dialysate reflective of fluctuations in the brain. Microdialysis is highly versatile, with its sensitivity, selectivity, and number of analytes that can be monitored simultaneously dependent on the detection method employed. Its main limitation is spatiotemporal resolution, as microdialysis probes are typically at least 200 *μ*m in diameter, and samples are historically collected approximately every 5–20 min to allow sufficient sample volume accumulation at low flow rates.^5–8^ Recent improvements, largely due to reduction in the minimum volume needed for sample analysis, have permitted microdialysis measurements on a subminute time scale.^9–12^

For the subset of brain molecules that are electroactive, particularly biogenic amines, electrochemical monitoring has flourished as an alternative methodology.^13,14^ This approach was first attempted in the Ralph Adams lab with a carbon paste electrode implanted in the striatum of an anesthetized rat.^15^ Slow potential sweeps between −0.2 to +0.6 V vs Ag/AgCl revealed peaks in current corresponding to the oxidation and reduction of electroactive substances; however, the identity of the mole-cule(s) producing the signal was unclear, with the authors suggesting it could arise from dopamine, norepinephrine, or ascorbic acid. Indeed, early voltammetric measurements suffered from poor chemical resolution between catecholamines and other easily oxidized species, often present in the brain at higher concentrations.^15–20^ In response to these problems, criteria were developed to ensure that intended analytes were indeed the source of recorded signals, including electrochemical, anatomical, pharmacological, and independent verification.^21–23^ A major advance to the field came with the development of fast-scan cyclic voltammetry (FSCV), a technique that utilizes rapid potential sweeps to oxidize and reduce analytes of interest.^24–26^ This process produces cyclic voltammograms, which display measured current as a function of the applied potential, that serve as “fingerprints” for compound identification, providing an advantage over single potential techniques.^26–28^ This moderate chemical selectivity allows the use of chemometric methods to separate, and subsequently quantitate, analytes with different current–potential characteristics (see Chemometric Data Analysis section below).^28,29^

The development of carbon-fiber microelectrodes (CFMs) has aided the FSCV field in multiple ways.^30–32^ The small size, and thus reduced capacitance and time constant, of these electrodes permits rapid scan rates (>100 V/s), which enables measurements on a subsecond time scale. Additionally, this relatively small size compared to traditional probes increases spatial resolution and permits localized measurements in discrete brain regions. Moreover, in contrast to tissue damage observed near microdialysis probes, minimal damage is seen surrounding fiber implantation sites.^33,34^ These probes are also easily modified with a variety of surface coatings, which can improve chemical selectivity, electron transfer kinetics, and sensitivity.^35–39^ Lastly, carbon-based electrodes demonstrate strong biocompatibility, and are more resistant to biofouling than metal electrodes. These advantages make FSCV with CFMs an attractive measurement technique for rapid neurotransmitter dynamics.

## Development of FSCV for Freely Behaving Animals

Measurements using FSCV with CFMs were originally conducted in anesthetized animals.^25,26,40,41^ However, these studies could not reveal direct information about neurotransmission during behavior. The first FSCV measurements in freely moving animals detected dopamine release in terminal regions, evoked by electrical stimulation of afferent axonal pathways in rats. These experiments used acutely implanted glass-encased CFMs lowered into the brain using head-mounted microdrives.^42–44^ Later, behavioral evoked dopamine was detected by this approach,^45^ and these types of recordings became routine, primarily due to improved sensitivity obtained by increasing the anodic limit of the waveform^46,47^ to maintain oxygen-containing moieties on the electrode surface which enhance adsorption of positively charged analytes (such as dopamine).^27^ FSCV has been adapted for multimodal recordings with simultaneous extracellular electrophysiological recordings^48–51^ and iontophoresis^51–54^ at the same probe.

The most recent generation of FSCV use in behaving animals has been to adapt CFMs for chronic implantation, permitting longitudinal measurements over an extended time scale in the same animal. This is not a novel direction for electrochemical monitoring, as earlier methodologies had adopted such an approach.^55–57^ These papers, which utilize amperometry, are notable because they clearly show the need for more chemical specificity in the measurements due to difficulty assigning the source of the signal. The standard fabrication of CFMs for FSCV using a glass-encased design had limited success when chronically implanted.^58^ However, the chronic CFMs used today employ a basic design where a carbon fiber is sealed in a small diameter fused-silica tube.^59^ Similar to results at acutely implanted CFMs^33^ and other miniaturized devices,^60^ these electrodes were demonstrated to avoid the progressive immune response and cell death that can impair measurements at larger probes.^34^

With the improved sensitivity^27^ and low-noise^61^ of modern approaches to using FSCV in vivo, recordings in striatal regions permit detection of dopamine elicited by task-related events such as the delivery of primary rewards,^62,63^ including pharmacological rewards,^29,64–67^ or reward-associated stimuli.^51,62,68–70^ In addition, spontaneous dopamine “transients” (i.e., brief elevations in extracellular dopamine concentration above the ambient level, produced by release events) can be observed. These do not appear to be time locked to overt stimuli^71–73^ but are dependent upon activity in the ventral tegmental area,^74^ and have been suggested to be a contributor to ambient extracellular dopamine levels in the nucleus accumbens.^75^ While the function of these dopamine transients has not been fully characterized, their activity can be altered by behavioral context^73^ as well as pharmacological agents including drugs of abuse.^64,71^

The objective of this paper is to discuss the nuances of using FSCV in behaving animals, based primarily on experience on measuring striatal dopamine. We will attempt to discuss the potential pitfalls that can make the use of FSCV or related approaches challenging, and then summarize how these caveats differentially affect alternative approaches, with a particular focus on the use of acute or chronic electrodes.

## Experimental Considerations

### Electrode Materials and Design

The most common construction of recording electrodes for FSCV in behaving animals uses carbon fibers housed in glass or fused-silica capillaries. These carbon fibers host surface moieties, such as carbonyl, hydroxyl, or more complex groups,^76,77^ which can alter the electrochemical properties of the carbon fiber by changing its surface charge and steric properties. The constellation of functional groups on the carbon surface can be tuned with electrochemical,^78^ thermal,^79^ or chemical^80^ pretreatment, and will determine the selectivity of the adsorption of molecules to the surface, including fouling agents and electrochemical analytes. A popular approach using FSCV at CFMs is to use the applied waveform on each FSCV scan to electrochemically condition the electrode, essentially “pretreating” the electrode surface each time a measurement is made.^47^ Specifically, increasing the anodic limit of the waveform above 1.0 V versus Ag/AgCl substantially enhances the sensitivity to dopamine by increasing dopamine-adsorbing oxide groups on the carbon surface and slowly etching the fiber surface to mitigate the effects of irreversible fouling.^27,47,77^ This approach has the advantage over traditional pretreatment strategies in that equilibrium is maintained throughout the experiment, providing stable sensitivity.

**Figure 1:**
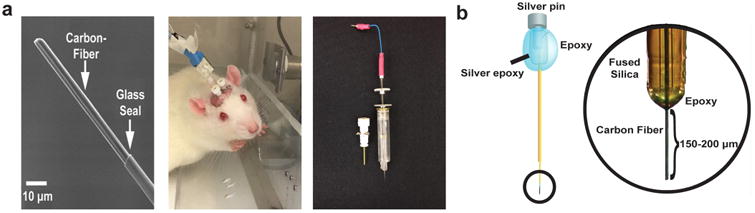
Designs of (a) borosilicate glass (b) fused silica CFMs. (a) Carbon fibers are aspirated through borosilicate glass under vacuum. A seal is created by heating and pulling the capillary to a fine tip. The protruding fiber is then trimmed, typically between 75 and 125 *μ*m. For optimal electrochemical performance, epoxy resin is used to fill any leaks in the seal that occur during electrode fabrication. Left panel: Electron micrograph of CFM. Reprinted with permission from ref 113. Copyright 2003 American Association for Clinical Chemistry. Middle panel: A rat with dual cannulas for later acute implantation of a CFM and reference electrode. The rat is tethered to a swivel and commutator via fastening of the headstage to an implanted stimulating electrode. Right panel: Side view of cannula for acute implantation of electrodes (left) and a micromanipulator for precise driving of the CFM during in vivo recordings (right). (b) Carbon fibers are threaded through a small diameter fused silica capillary under isopropyl alcohol. After drying, epoxy is placed on the fiber and wicked into the fused silica capillary to create a hemispherical seal (inset image). The protruding carbon fiber is trimmed between 150 and 200 *μ*m long. Electrical connection is established between a silver pin and the fiber with silver epoxy, which is later insulated with clear epoxy. Reprinted with permission from ref 59. Copyright 2010 Nature Publishing Group.

Construction of CFMs involves housing a carbon fiber in a capillary insulator with an exposed length at one end (the sensor) and an electrical connection at the other. For glass-based electrodes, a single carbon fiber is aspirated into a borosilicate glass capillary (600–1000 *μ*m outer diameter, 400–500 *μ*m inner diameter) (Figure 1a, left panel). The capillary is then pulled on a commercial glass-electrode puller (either vertical or horizontal) to produce a tapered seal onto the carbon fiber. Sometimes the glass seal is then deliberately broken and resealed using epoxy (Epon 828 with 14% *m*-phenylenediamine by weight). This approach increases the robustness (i.e. prevents unintentionally exposed carbon fiber from providing a low resistance path for current) and reduces the shunt capacitance of the electrode taper by providing a thicker insulating layer between the carbon fiber and the extracellular fluid. For fused-silica-based electrodes, a single carbon fiber is loaded into a polyimide-coated fused-silica tube (90 *μ*m outer diameter, 20 *μ*m inner diameter, 8–12 mm length) submerged in isopropyl alcohol (Figure 1b). With the carbon fiber protruding, one end of the tube is sealed with epoxy (Devcon 20845). For either electrode type, the carbon fiber protruding from the seal is then trimmed to the desired length, and an electrical connection is made at the other end. Lengths of the trimmed carbon fiber usually range from 50 to 200 *μ*m, where longer exposed fibers are more sensitive, but have lower spatial resolution. Typically, glass-based electrodes have been used for acute implantation, while fused-silica-based electrodes are favored for chronic implantation due to both their durability, and biocompatibility arising from their narrow diameter and polyimide coating.^81^

Not all electrodes are created equal. For glass electrodes, the structural integrity of the pulled seal is the most important determinant of electrode performance. Large cracks or gaps in the seal will lead to exposed fiber and/or increased fragility, which will impede electrochemical measurements. These problems can be alleviated by the use of epoxy to reinforce the seal (see above). For fused-silica encased CFMs, it is important that no epoxy remains on the fiber itself and that the seal forms a convex, rather than concave, seal (see Figure 1b for illustration). Electrochemical characteristics of either electrode design can be tested pre-experiment, either in vitro (i.e., in buffer) or in vivo (i.e., during surgical implantation of chronic CFMs before cementation, or after lowering acute CFMs), to observe noise levels and ensure electrical connectivity.

### Experimental Design for FSCV Recordings in Freely Moving Animals

Measurements in freely moving animals are conducted using head-mounted amplifiers (“headstages”), which connect to the CFM and reference electrodes and transduce the experimental current into voltages for downstream data collection and analysis.^82,83^ These headstages are anchored to the animal's heads either directly via an electrical connector or at a separate point, such as the pedestal for the stimulating electrode assembly (Figure 1a, middle panel). The headstage is also connected to a swivel and commutator that permits movement within the behavioral chamber. Depending on the type of electrode used, cannulae may be affixed to the skull for later implantation of fresh CFM or reference electrodes (Figure 1a, middle panel) or fused-silica CFMs can be cemented directly to the skull.

The electrode design used influences both the type of experimental questions that can be answered and the overall success rate of recordings. The fragility of borosilicate glass electrodes can lead to a lower yield of successful experiments with respect to fused-silica implantations. Moreover, tissue damage from repeated insertion limits the number of within-subject recordings.^42,83^ Once successfully lowered, however, these electrodes tend to be stable over the course of individual measurement periods. Thus, these electrodes are best served for studies in which experimentally relevant manipulations occur during single recording sessions.

Conversely, the flexibility of fused-silica electrodes permits a higher success rate for implantation compared to glass electrodes. For chronic recordings, fused-silica electrodes are affixed to the skull with dental cement and left unused for at least 1 month to allow the immune response to these probes to dissipate.^59^ Following this waiting period, it is possible to conduct many recordings at each electrode, increasing the data yield of chronic electrodes over acute electrodes. Longitudinal measurements permit the monitoring of dopamine over extended behavioral training and treatments. This is particularly relevant for models of disease states in which conditions develop slowly over time.^84^ These electrodes are routinely used for periods up to 4 months of recording. Naturally, there is some attrition of usable electrodes over that time. The majority of this attrition pertains to physical failures (e.g., separation of surgical implant from subject's head, loss of electrical continuity) and is much more infrequently due to altered electrochemical properties of the electrode (see Figure 3a, Supplementary Table 1 of Clark et al.^59^).

### In Vivo Electrode Positioning

**Figure 2:**
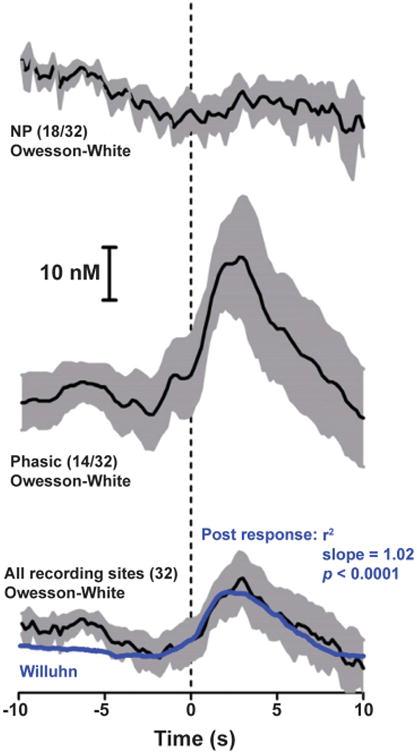
Comparison of concentrations measured at acute and chronic CFMs without optimization for dopamine release sites. In a study with acute CFMs (Owesson-White et al., ref 87), electrode placement was optimized for extracellular electrophysiological signals rather than dopamine release, resulting in recording locations without (top) and with (middle) phasic dopamine release. The concentration profile corresponds well with values from chronically implanted CFMs that were not optimized for recording location (bottom), indicating the lower concentrations measured with chronic CFM may be a result of recording site selection. Reprinted with permissions from ref 86. Copyright 2012 PNAS.

**Figure 3:**
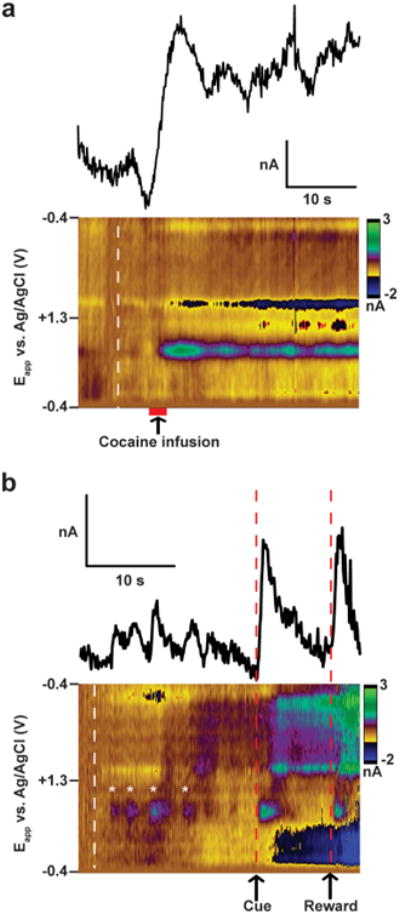
Dopamine transients at chronically implanted CFMs. (a) Pharmacologically induced dopamine transients at a chronic CFM in response to i.v. cocaine infusion (red bar, 1.5 s duration). Background subtraction is denoted by the white dashed line. (b) Measurements at a chronic CFM during a behavioral session of Pavlovian conditioning. Spontaneous dopamine transients are observed preceding cue onset (white asterisks). Moreover, both cue onset (left red dotted line) and reward delivery immediately following cue offset (right red dotted line) evoked phasic dopamine release. Background subtraction is denoted by the white dashed line. Dopamine traces were extracted with PCR using a standard training set. Both measurements were made in the nucleus accumbens core.

For different applications, recording electrodes can either be fixed in the brain, or can be housed in a microdrive that allows their position to be adjusted (Figure 1a, right panel). The former is amenable to multiple electrodes in the same animal,^59^ whereas the latter permits systematic mapping of heterogeneity of electrically or naturally evoked dopamine release along the dorsal-ventral axis.^85^ Microdrives also allow selection of a recording site within this heterogeneity by identifying “hot spots” (i.e., areas with a high density of release sites).^73,85^ Placement of fixed electrodes does not typically use this type of feedback-based selection. Therefore, positioning of these electrodes is more akin to random sampling of the tissue, and so signals converge upon the population average rather than local maxima. Consequently, signals measured with fixed electrodes tend to be smaller than those from drivable electrodes due to unbiased selection of recording sites (Figure 2).^86,87^ Dopamine signals in regions without release or uptake sites rely on diffusion from nearby terminals, and these sites exhibit both slower rises and decays compared to “hot spots”.^88^ As a result, electrodes not deliberately targeted at regions of high terminal density would be expected to have slower signals due to heterogeneity of release sites.^85^ For similar reasons, one would intuit that fixed electrodes should detect fewer spontaneous transients. However, most studies using chronic electrodes focus on the analysis of task-related events, and so spontaneous transients have seldom been reported. Nonetheless, on the rare occasion when they were quantified, they were comparable in detected frequency as those measured with drivable electrodes.^72^ Figure 3 demonstrates examples of pharmacologically and behaviorally evoked dopamine transients, as well as spontaneous transients, measured at chronically implanted CFMs.

Acute electrodes have the advantage of being drivable. However, in addition to the concerns with electrode fragility during repeated use mentioned above, electrode insertion imposes restraint stress on the animals. This could impact behavioral assays that study stress under controlled conditions.^89^ Because chronic electrodes do not require repeated insertion, they do not share these issues. Although chronic electrodes are not drivable in regular use, chronically implanted electrode arrays have been used that permit independent movement of electrodes within the array.^90^

### Reference Electrodes

Experiments using FSCV in freely moving animals generally use chronically implanted Ag/AgCl reference electrodes.^83^ An issue with this approach is that half-cell reaction is not maintained over time, producing a shift in the reference potential and polarizing the reference electrode, most likely due to dechlorination.^91^ Further, fouling of the reference electrode would be expected upon insertion. This status is evident from an altered shape of the background current.^92^ While the shift in reference potential can be compensated for by positive offsets to the applied potential, some nonlinearity may be introduced by the polarization if voltage error persists.^93^ Use of a polymer coating on the Ag/AgCl surface has been shown to delay dechlorination.^91,94^ Alternatively, reference electrodes can be implanted on the day of recordings through a guide cannula.^46,95,96^

### Signal Stability

During each voltage scan with FSCV, a cyclic voltammogram (CV) is generated that contains faradaic (redox) current from electroactive neurochemicals. In addition, there are other sources of current, primarily from the electrode itself, which produces both faradaic current from redox processes at its surface moieties, and nonfaradaic current due to its resistive-capacitive properties. The “background” current from the electrode is quantitatively much greater than the current produced by physiological levels of neurochemicals. For this reason, background subtraction is used with FSCV to measure changes in analyte concentration from a baseline reference point: CVs obtained during the baseline period are averaged and subtracted from each of the subsequent CVs in the time series. This approach allows the detection of bidirectional changes in the concentrations of electroactive neurochemicals from the baseline. However, any changes in the other components of the CV following the baseline period will necessarily also contribute to background-subtracted CVs. The electrode background current described above is quite stable from scan to scan, but because it dominates the CV, even very small changes in the electrode's chemical or physical properties following the baseline period can contaminate background-subtracted CVs in the form of “drift”.

The first type of drift we will discuss is that relating to the chemical properties of the electrode surface. This type of drift is most prevalent when applying waveforms to the electrode that have anodic limits that exceed 1.0 V versus Ag/AgCl. Application of these waveforms in aqueous solutions such as the interstitial fluid in the brain, changes the surface chemistry of carbon fibers by introducing surface oxide groups,^77^ increasing the faradaic current in the CV. Until this “activation” process reaches equilibrium, there will be progressive increase in the overall current in the background CV, as well as a net negative potential shift in the background peak.

To get to equilibrium more expediently, waveforms can be applied (“cycled”) at a higher repetition rate than that used for data collection (typically 60 Hz). The required time to reach equilibrium differs across electrodes and implantations. In practice, acutely implanted electrodes are cycled for 15–30 min at 60 Hz before use. Chronically implanted electrodes are typically cycled more extensively, as much as 2 h on the first use, followed by shorter durations (30–60 min) for each subsequent recording. As the necessary amount of cycling to reach equilibrium can vary between electrodes, however, it is more reliable to assess electrode stability via the background CV, which should remain relatively consistent in shape and amplitude following cycling. With either approach, additional cycling at the data-collection repetition rate (usually 10 Hz) for at least 10 min is required to re-establish equilibrium at this waveform application frequency. Nonetheless, even with extensive cycling of the electrode before the experiment, some drift may still persist.

Another type of background-current drift can be caused by etching of the carbon fiber during voltage scans. Etching drives evolution of the electrode surface and thereby affects both the faradaic and nonfaradaic currents. The extent that an applied waveform will produce etching of carbon fiber is dependent on its duration at higher potentials, specifically the period in which the applied potential remains greater than 1.0 V versus Ag/AgCl.^97^ With the waveforms typically used in FSCV for in vivo dopamine detection, the excursion above 1.0 V is relatively short (1.5 ms/scan) and so, any etching that takes place is incremental over millions of scans (∼0.002 Å/scan).^97^ Therefore, drift attributable to this process occurs at a much lower rate than that from changes in surface chemistry. Thus, two main sources of background drift are augmented using voltage waveforms that that have an anodic limit in excess in 1.0 V. This drift is a trade-off with the increase in sensitivity afforded by these waveforms.^27^

The structural quality of the electrode and its connection to the headstage can also impact the stability of the signal. For example, if the seal between the carbon fiber and the insulating capillary is compromised then fluid can leak into the capillary increasing the background size (i.e., producing drift). The likelihood of this problem occurring can be reduced using epoxy to make, or reinforce, the seal. The integrity of electrical connections between the electrode and headstage are also important, especially with regard to movement artifacts. These types of problems are largely eliminated with practice in electrode fabrication, combined with robust quality control prior to implantation.

These instabilities in the signal can interfere with reliable signal analysis. While the reduction of noise can lessen this issue (e.g., with good electrode quality control), background drift poses a particular problem. Background drift, by definition, is an accumulative process where the level of interference in an analytical signal increases from the baseline (subtraction) period, limiting the effective window of analysis. Heien and colleagues suggested, as a guideline, that with standard parameters for FSCV in behaving animals, chemometric data analysis (see below) remains reliable for CVs taken up to 90 s from the baseline.^29^ This window is sufficient for the routine use of peri-event histograms to test changes in analyte concentration time locked to a stimulus or action. However, as discussed below, the exact size of a reliable analysis window will be dependent upon the quality of the data, and will be assessed as part of the data-analysis process. To attempt to remove the influence of drift, thereby increasing the analysis window, one strategy has been to incorporate CVs representing the drift in to the training sets used for analysis.^98,99^

When considering different types of electrode with respect to signal stability, a number of factors come into play. Glass-based electrodes are more fragile than fused-silica-based electrodes and are therefore more susceptible to noise from compromised seals or other structural damage. Fixed electrodes have a low profile with connectors cemented in place, reducing movement artifacts and overall noise due to the absence of pendulum effects from a microdrive, or movement of wires relative to the electrode and headstage. However, these electrodes cannot be easily replaced with a fresh electrode in the event of a failure. Drift relating to the surface chemistry of the electrode is dependent on the type of carbon fiber used and the waveform applied. These aspects are not systematically different between acute and chronic electrodes and so neither application appears to be more susceptible to this type of background drift.

By the same rationale, the *rate* of background drift due to etching should not differ between acute and chronic electrodes. However, because the cumulative duration of recording with chronic electrodes is substantially longer than for acute electrodes, it is likely that the *total* etching across the working lifetime of a chronic electrode will be greater. This may impact the sensitivity of the electrode. For this reason, it is advisable that positive controls are used to ensure that the sensitivity is not changing over the course of an experiment (e.g., Figure 2E of Clark et al.^100^).

## Chemometric Data Analysis

Extracellular dopamine is detected via its oxidation and reduction at the carbon-fiber surface, producing a voltammetric current proportional to its local concentration. However, how to obtain this concentration has been a matter of considerable debate and development within the field. Original calibrations of in vivo voltammetric data directly converted the voltammetric current at the peak oxidation potential for dopamine into a concentration using an externally obtained calibration factor. However, various electroactive substances can interfere at the oxidative peak for dopamine, including ascorbic acid,^26,35,101,102^ dopamine metabolites,^17,26,102,103^ pH,^80,104–107^ and other ions.^104,107^ Because this method is univariate (i.e., only uses a single measurement point to predict concentration), it cannot separate out these interferences.^108,109^ While anatomical and pharmacological criteria can increase confidence in the identity of the measured signal, univariate analysis will fail if interfering analytes significantly contribute.

To circumvent this problem, a method was developed to compare experimental CVs to electrically evoked templates collected at the same electrode.^23,27,83,110–113^ CVs with a lower correlation coefficient than a user-defined value (typically *r*^2^ < 0.75) were considered to have significant contribution from other electroactive substances and were not used for univariate prediction. In some cases, current contributions from pH^111,112^ and drift^114^ were manually subtracted by using currents from a potential where dopamine did not contribute to predict current interference at the peak oxidation potential for dopamine. However, this approach can miss dopamine events that are identified with more rigorous analysis.^29^

**Figure 4:**
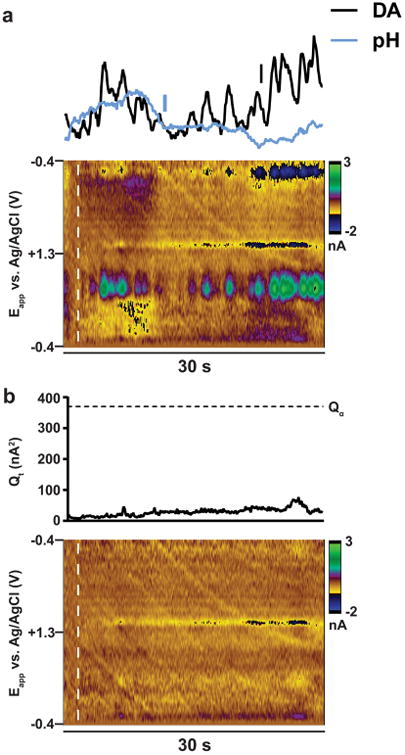
Example of the use of principal component analysis to analyze cocaine-induced dopamine transients. (a) A 30 second color plot following cocaine (20 mg/kg) administration in an awake rat shows overlapping dopamine and pH changes. The dopamine (black) and pH (blue) changes are separated by PCA, and quantitated using inverse-least-squares regression. pH changes have a maximum contribution of +0.019 pH units (−0.76 nA) at 8.3 s, while dopamine maximizes at 262 nM (3.13 nA) at 28.9 s. (b) Residual analysis confirms that the PCA model is valid for analysis of this data. *Q*_t_ values (black) fall below the model specific tolerance level (*Q_α_*, 379 nA^2^) for the data shown in panel (a). A residual color plot displays current uncaptured by the model.

A more reliable calibration methodology is the use of chemometric multivariate analysis. Instead of using measurements at a single potential to predict concentration, multivariate analysis uses the entirety of the potential window to separate and quantitate multiple analytes, taking advantage of the chemical selectivity afforded FSCV (Figure 4a).^115–117^ While there have been a few different multivariate methods implemented with in vivo FSCV data,^118,119^ the most implemented and characterized method with FSCV data is principal component analysis (PCA) with inverse least-squares regression, also referred to as principal component regression (PCR).^28,29,120–123^ Therefore, the focus of this section of the Review will be on the use of PCR for analysis of FSCV data. Nonetheless, the fundamental theory behind PCR is similar to other multivariate methods.

### Principal Component Regression

Data collected with FSCV tends to be complex. At high sample rates (>100 kHz), there are approximately 1000 data points per individual CV. One of the chief goals of PCR is to reduce the dimensionality of data. In this way, a large number of data points can be described by a handful of abstract vectors referred to as “principal components” (PCs). Despite this reduction in dimensionality, PCR extracts more information from the data than univariate methods, and allows resolution of simultaneously varying analytes with overlapping signals.^29^ PCR also functions as a noise removal technique, because PCs that represent nondeterministic variance (i.e., random noise) in the training set are discarded. This process improves the quality of its determinations and allows stronger confidence in the model. Lastly, this method provides objectivity and statistical validation of the measured signal.

Generally, the construction and application of a PCR model to predict concentrations from FSCV measurements consists of five steps: (1) training set construction, (2) generation of PCs, (3) discarding PCs that only represent noise (i.e., rank determination), (4) signal extraction, and (5) model validation. Importantly, free software (HDCV) is available that automatically carries out steps 2–5 and is compatible with data collected with TarHeel and other voltammetric software.^124^ This software also includes additional diagnostics to assess training set quality. Nonetheless, it is important to understand the basic concepts of PCR to use it effectively. With this aim in mind, each step will be described briefly. More detailed discussion of PCR^115–117^ and its use with FSCV is available elsewhere.^28,29,120–123,125,126^

#### 1. Training Set Construction

The first step in building a PCR model is the collection of a group of CV standards known collectively as a “training set”. Several guidelines for building training sets have been outlined previously.^115,120–123,125,126^ First, the training set should comprise all expected contributions to the data. For measurements of striatal dopamine, this typically includes dopamine and pH changes, though background drift has also been included.^98,99^ Second, the CV standards should span the expected current range in the data to be analyzed, which prevents model extrapolation.^115^ Third, the training set should contain an adequate number of samples. While there is no strict consensus on the ideal number of standards, a minimum of three standards per analyte is needed to satisfy the requirements for regression.^115^ The use of a larger number of standards is preferred, however, and previous work has suggested that five CVs per analyte is sufficient to provide reliable models.^29,115,122,123^ Fourth, to satisfy mutual independence, training set CVs should be selected from separate events, and not include CVs that will be analyzed by the final model. Finally, a training set should be generated in a recording environment that matches the experimental environment.

#### 2. Principal Component Generation

Next, the training set standards are used to generate the PCs. As such, the quality and representativeness of training set standards is of critical importance. Importantly, the largest amplitude standards in the training set dominate the appearance and quality of these PCs; this is because FSCV standards are not usually mean-centered to avoid giving undue influence to the smallest standards in training set CVs, which typically have the lowest signal-to-noise ratio.^115^

These PCs are determined by singular value decomposition (SVD), a process that is described in detail elsewhere.^126^ With SVD, each successive PC is calculated to span as much of the remaining variance in the training set standards as possible. The maximum number of PCs for a particular model is equal to the number of measurements being made (i.e., for CVs with 1000 data points, there could be a maximum of 1000 PCs). However, the use of SVD limits the number of PCs to the total number of standards in the training set. The PCs were created from the same dimensions as the data, and thus can be visualized in the form of CVs.^122,126^ However, it is important to understand that PCs are by definition abstract, and thus should not be viewed as representing individual analytes.^120^ Indeed, it is extremely unlikely that PCs will precisely align to individual analytes because of the requirement of orthogonality between PCs in the PCA approach.

#### 3. Rank Determination

While several PCs are provided by SVD, only a subset has information that is relevant to concentration prediction.^122,127^ These are *primary PCs*, which represent analytically relevant variance in the standards, while *secondary PCs* reflect any remaining variance (i.e., noise). The number of primary PCs is referred to as the *rank* of the PCR model. The exclusion of secondary PCs is desirable, as it prevents the use of noise in concentration prediction and allows for an estimation of noise levels for model validation (see below).

Rank selection in FSCV is customarily done with Malinowski's F-test.^122,127^ his procedure is objective, statistically validated, and does not require pre-existing knowledge of noise levels in the data, which can be difficult to obtain. Moreover, it has been demonstrated to discard more noise than other methods.^122^ This process is most suitable for training sets with a signal-to-noise ratio larger than 10.^128^ Rank tends to increase when there is more variability between training set standards (i.e., peak shifting and broadening). Therefore, while a rank of two maybe desirable for a moderate training set size (e.g., 10 total standards) representing a two component system (i.e., dopamine and pH changes), the rank will vary both with the consistency of the CVs and the signal-to-noise of the training set.^122,126^

#### 4. Signal Extraction

The generated PCs are then used to extract concentrations of any analyte that was included in the training set. The first step is using the training set standards to generate “scores”, which are the dot products of each PC with each training set standard. Notably, CVs have higher score magnitudes with PCs they closely resemble in shape. Scores arising from secondary PCs are discarded, as these PCs describe only noise. The concentrations are then regressed against retained scores, producing a regression that defines the calibration model. To predict the concentrations of experimental CVs, their scores are determined for the retained PCs and plugged into this regression equation. This entire process has been depicted visually (see Johnson et al.^126^).

#### 5. Model Validation

As Douglas Adams states in *The Hitchhiker's Guide to the Galaxy*, “we demand rigidly defined areas of doubt and uncertainty”.^129^ Because multivariate calibrations are complex, it is important to verify that these models are of sufficient quality to capture experimental data and demonstrate what data remains uncaptured. This process is referred to as “model validation”. In other fields (e.g., spectroscopy), validation is performed by running independent standards on the instrument to determine the accuracy of the model.^115^ However, this is not possible when building an in vivo training set (see below), as the concentrations of analyte signals are not known. Therefore, a “pseudovalidation” procedure is applied to PCR analysis of FSCV data in which the ability of the model to capture the experimental data is assessed.^120,121,130^ In other words, this validation procedures tests the *applicability*, rather than the accuracy, of the model. Nonetheless, if the model is considered “invalid” (i.e., not applicable) for a particular experimental datum, the concentration value obtained is rejected.

One method for evaluating model validity relies on the “first order advantage” of multivariate calibration, which allows for detection, but not removal, of interfering signals through residual analysis.^108,109^ During PCR, primary PCs are used to reconstruct experimental CVs. However, it is rare for these PCs to fit the data perfectly, with remaining uncaptured current referred to as the “residual”. Jackson and Muldholkar developed a procedure to statistically test residual values to validate the model.^130^ A significance threshold is determined using the secondary PCs that were discarded during rank selection at a user-defined confidence interval *α* (*Q_α_*), under which 100 × (1 – *α*)% of uncaptured random noise should fall.^120,121^ the squared sum o the residual current for a particular CV (*Q*_t_) is greater than *Q_α_*, it is determined that a significant current source is present that cannot be captured by the model, which invalidates its use for analysis of this data. The concern with deterministic variance being present in the residual is that this variance may be the result of misattribution of dopamine signal to the residual rather than the dopamine vector (false negative). Alternatively, it could be an indicator that a signal that is not identical to dopamine is attributed to the dopamine vector (false positive) since the remainder of that signal (i.e., the difference between the CV for the signal and that for dopamine) would be attributed to the residual. However, the source of deterministic variance could also be due to ancillary noise sources such as an unexpected electroactive neurochemical or movement artifacts in that absence of false positives or negatives. Therefore, the process is conservative inasmuch as the model will be rejected if *Q*_t_ exceeds *Q_α_* because of false-negative or false-positive errors, but also due to the presence of other components that cannot be accounted for by the model. Importantly, this process does not statistically confirm whether the collected data contains dopamine; this can only be confirmed with pharmacological and/or histological tests, or selective (i.e., optogenetic) stimulation.

The residual (*Q*_t_) is calculated for each CV in any given set of data, and these values can be plotted along the same time scale as the data (Figure 4b, top). Residual color plots can be used to visualize uncaptured current, which could reveal the source of variance uncaptured by the training set (Figure 4b, bottom). Residual failure outside of the time window in which concentrations are being predicted should not impair the ultimate success of the model. However, *Q*_t_ may cross *Q_α_* for multiple CVs within the prediction window (i.e., during prolonged dopamine and/or pH events). Any individual data point that fails residual analysis (i.e., *Q*_t_ > *Q_α_*) is excluded from the data set. The omission can be executed by replacing the data point with a new value based on interpolation between adjacent data points, or by designating the data point as “NaN” (not a number). Additional a priori exclusion criteria are also utilized if there are too many data points missing from a trace that represents the single unit of analysis (e.g., one trial around a task-related event). Any trial is removed from subsequent analysis if it fulfills *either* of the following two criteria: (1) a total of 10% or more of the data points have been excluded, or (2) a string of contiguous data points have been excluded that amounts to more than five percent of the data points.

### Additional Diagnostics to Test the Quality of Training Sets

Further procedures are available to assess the quality of training sets. One such tool is a Cook's distance plot, which displays the scores for each analyte of interest with respect to the primary PCs.^123^ For the sake of simplicity, these are typically depicted with the *x*- and *y*-axes representing the first two primary PCs, though for higher dimension models (i.e., training sets with a rank > 2), it should be understood that more projections exist. The use of these plots, along with calculation of Cooks' Distances, also allows the identification of outliers in the training set, described elsewhere.^123^

The robustness of a training set can also be assessed with the model k-vectors (sometimes referred to as a K-matrix). A k vector is typically calculated to represent the estimation of the CV for a pure unit analyte concentration change (i.e., 1 *μ*M dopamine or a full pH unit change).^123,126^ A representative k vector indicates the success of the model in isolating analytes of interest from the training set standards. A k vector that does not resemble the desired species can arise from the poor quality of training set standards and/or significant differences between them.^123,125,126^ Notably, it has been shown that the quality o CVs for each analyte (i.e., DA and pH for typical training sets) can affect the predictions for the other analytes in the training set, making the quality of standards for each analyte in the training set an important experimental aim (in particular, see Figure 1 of Keithley and Wightman^123^).

### PCR with Residual Analysis in Practice

The most controversial component of the chemometric PCR analysis for FSCV is the construction of a training set.^125,126^ The standard procedure for constructing a training set for chemometric analysis is to use a series of known concentration standards applied to the instrument in vitro, for example in a flow cell. However, even when collected at the same electrode, in vivo and in vitro CVs differ (see Supplementary Figure 3 of Clark et al.^59^). This is likely due to chemical and electrical (impedance) differences between the two environments^23,29^ and has led to the practice of acquiring training sets in vivo by stimulation of an afferent dopamine pathway following the experiment,^29,123,125^ which is an extensively characterized source of dopamine release in vivo.^24,29,41,131^ This stimulation evokes both dopamine release and a subsequent temporally resolved hemodynamic response, including a pH change.^112^ Notably, these pH changes are difficult to resolve from changes in other electroactive substances (e.g., H_2_O_2_/O_2_, adenosine) that also occur in response to electrical stimulation. As a result, in vivo pH CVs typically include contributions from these substances, and are thus difficult to simulate in vitro.^132^ Ultimately, with a series of stimulation intensities (e.g., current amplitude, pulse number, frequency), a training set can be constructed that spans the range of signals from dopamine and pH observed under experimental conditions. This method produces CVs that match the electrochemical and biological environment of the data to be analyzed, which is important for PC generation, signal extraction, and residual analysis (see above).

However, unlike a training set generated from exogenous standards, the analyte concentrations producing these in vivo signals are not inherently known. Therefore, to estimate the concentrations of analytes in the training set, an additional step is required. Common practice is to use in vitro standards to obtain a calibration factor to convert current to concentration. Thus, while the analyte identity is not determined from in vitro standards, the estimation of concentration is. As stated above, in vitro CVs do not perfectly map onto in vivo CVs and, as such, the mapping of a calibration factor also incorporates some level of inaccuracy. For this reason, it is important to recognize that analyte concentrations reported from in vivo FSCV experiments should be regarded as estimates.

An additional limitation of the use of in vivo training sets is that, rather than using chemical standards, biological signals of presumed chemical origin are employed. Therefore, under these conditions, the model extracts signals that are similar to those produced by the biological manipulation rather than signals that are necessarily specific to a particular chemical. This approach is tolerated as a proxy of a chemical signal when the signal evoked by the stimulus used to generate the training set has been well characterized (such as in vivo stimulation along the ascending dopaminergic pathway, discussed above).

Using the original incarnation of chememotric analysis of in vivo FSCV signals,^29^ training sets and experimental data are collected from the same recording site (or sometimes at different recording sites from the same subject), and thus lack full statistical independence. In these cases, the model identifies signals at a recording site evoked by one stimulus that resembles signals at the same recording site evoked by a different stimulus; or even by the same stimulus when electrically evoked signals are analyzed using a training set generated from electrical stimulation at the same location.^46,133–135^ One means utilized to avoid this circularity, and obtain greater independence, has been to construct in vivo training sets in a different subject to that from which the experimental data will be collected. However, for practical (and ethical) reasons, it is not always possible to take each electrode used in an experiment and collect a training set within a separate animal. Consequently, the use of “standard” training sets has evolved where a model is built from a training set generated at one electrode and used to analyze data from another electrode in a different subject. This approach assumes generalization of signals across electrodes. Indeed, electrochemical detection is founded on the premise that molecules exhibit consistent faradaic properties on a particular substrate when conditions are reproduced. Therefore, the key to the success of this approach is to maintain reproducibility of electrode fabrication, a goal that may be more favorable for (non-pulled) fused-silica than for pulled borosilicate-glass based electrodes, which tend to have significant variation in their tapers. However, other sources of variability can also violate generalization across experiments, including reference electrode drift and electrochemical differences between different carbon fibers.

There are some additional advantages to using standard training sets. Models no longer need to be built at each individual electrode, which results in reduced analysis time. In addition, the use of a single standard training set could avoid the variability between experimenters in training set construction that has been demonstrated previously.^122^ Finally, a stimulating electrode, which could perturb the tissue and ultimately affect behavior,^136^ does not need to be implanted in the experimental animal.

Nonetheless, there are limitations to this approach. The ultimate characteristics of any PCR model are dependent on the CVs provided for the training set, and not the data to which it is being applied. The primary PCs, those used for concentration prediction, will exhibit characteristics of the signals seen at whichever electrode was used for training set construction. If there are differences in CV shapes between the experimental data and the training set, primary PCs will prove less able to extract and attribute experimental currents to the desired analytes. A recent study demonstrated that CVs differ between electrodes and experiments and, despite high correlation coefficients between k vectors, this leads to differences in predicted dopamine concentrations.^125^

A more significant problem is the impact on the reliability of model validation. Differences between experimental and standard training set CVs may lead to the assignment of deterministic currents (i.e., signals arising from analytes in the training set) to *Q*_t_, resulting in unrepresentative residual traces. In some cases, this can lead to data being discarded that would have been retained with a within-subject training set (i.e., a false negative).^125^ Moreover, because *Q_α_* is determined from information in secondary PCs, it will be model-specific and invariant across different sets of data even when noise levels vary from experiment to experiment. Lower than expected *Q_α_* values could increase the rate of false negatives; however, unrepresentatively high *Q_α_* values are also possible, which could lead to the retention of data that should have been discarded (i.e., false positives). This is more concerning, as it would permit the retention of poor data.

**Figure 5:**
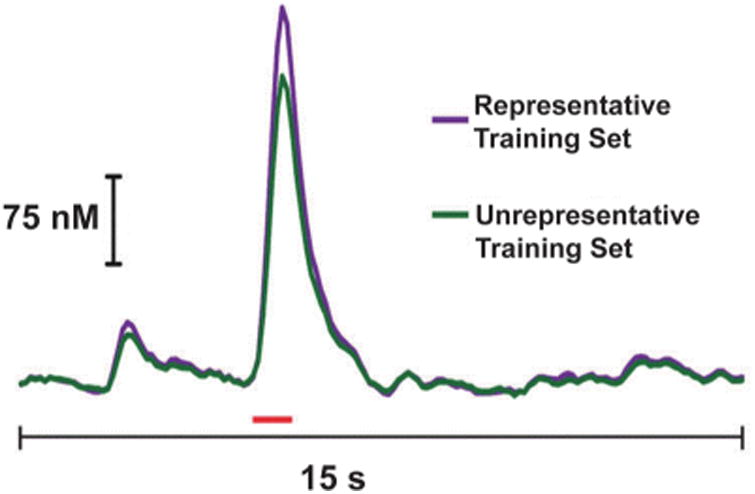
Training sets built with data from separate electrodes could capture qualitative information. Dopamine CVs from a training set built at the same electrode as the collected data were replaced with dopamine CVs from a separate electrode, while pH CVs were left unaltered. Analysis with this composite training set resulted in underestimation of signal, but tracked qualitative information for this electrical stimulation (red bar). Reproduced with permission from ref 123. Copyright 2011 American Chemical Society.

Ultimately, standard training sets suffer from the disadvantage of being unrepresentative of the experimental data. Nonetheless, standard training sets could provide similar qualitative results to within-subject training sets. Previous work has demonstrated that replacing dopamine CVs in a within-subject training set with CVs from a separate electrode (leaving pH CVs unaltered) resulted in a qualitatively similar trace (Figure 5),^123^ and comparison of data from different experiments using within-subject and standard training sets, respectively, has yielded similar results (Figure 2). However, current standard training set methodology precludes the ability to test whether the quantitative or residual analysis failures outlined above occur for any given application. Thus, improvements to standard training set methodology to reflect these concerns are important. One method that has been adopted to provide a level of validation between the generalized model (standard training set) and the experimental data is to use positive controls at the start and end of the experiment to compare the evoked signals with those in the training set. Commonly for experiments where striatal dopamine is being recorded, the unexpected delivery of a food reward is used to elicit an electrochemical signal.^100,137^ This signal is then compared to the CVs in the training set. If there is poor correlation between the positive-control signal and the training set, then either the signal is not predominantly dopamine, or the model will not generalize to the electrode being tested. While one could not easily discern these two scenarios, in either case it would not be fruitful to continue to collect and analyze experimental data under these conditions. However, this procedure does not address similarity of pH signals or noise levels between the data and training sets, both of which influence the predictions and success of the PCR model. Therefore, improving this verification process is a warranted area for progress in future investigations.

In addition, the methodology for constructing and/or implementing standard training sets could be improved. Notably, multivariate calibration transfer between instruments or electrodes is a significant area of inquiry within the field of chemometrics.^138,139^ These methods often require independent standards being run on each instrument (not possible with in vivo measurements) or use data from the new instrument to update the model. Further collaboration between chemometricians and users of FSCV could improve standard training set methodology by incorporating differences between electrodes and instrumentation to better match the experimental environment.

### Guidelines for Methods Presentation

Because variability exists in procedures for PCR, a few basic guidelines for reporting these procedures are warranted. First, it is important to make clear what methods were used to construct training sets for the study. In particular, it should be elucidated which electrodes were used to generate the training sets (i.e., specific or standard training sets) so that readers can understand the procedure used to acquire and select standards. Second, because these chemometric models will be used to analyze large amounts of data, it is important to report their general characteristics. This would include the analytes that comprise the training set, *Q_α_* values, and rank. The use of k-matrices could also illustrate the quality of these training sets. Third, the criteria for exclusion of data (i.e., residual analysis) should be made clear. Lastly, the use of additional methods to increase confidence in the acquired signal (e.g., the use of positive controls to verify the applicability of the model to experimental conditions^100,137^) should be reported.

## Conclusions and Perspectives

The authors of this Review are in general agreement that, when appropriate caution is observed, both acute and chronic CFMs can be used for detection of behaviorally evoked dopamine release in regions of the striatum using FSCV. In support of the reliability of these measurements, there is generally high concordance between results from FSCV of dopamine concentration fluctuations in the striatum with either acute or chronic electrodes, and electrophysiological recordings of dopamine neurons in the midbrain, with many key findings reproduced across approaches. These replications include the characterization of reward prediction-error signals^62,70,140^ that convey quantitative information.^63,141^ They include demonstrations that dopamine signals to reward-related cues are sensitive to factors that influence subjective value such as delayed reward delivery (temporal discounting),^62,142,143^ or subjective risk preference,^144–146^ and concur that there is stronger encoding of reward size than effort-based response cost by dopamine signals.^147,148^ An uncertainty-like signal following presentation of a Pavlovian stimulus predicting probabilistic reward has been identified and replicated across methodologies^149,150^ as have observations of partial generalization between sensory stimuli that are associated with different economic values,^62,151^ which can come in the form of a presumed sensory signal, temporally separated from a value signal.^63,152,153^ The success of chronic electrodes is notable, as it has long been held that chronically implanted electrodes are prone to failure. A recent review of glucose biosensors documents the importance of chronic sensors for monitoring in diabetes. The chief problem to their use is the foreign body response that impairs sensor performance.^154^ It may be that the finding that very small electrodes remain functional will be very useful to other health related fields involving biosensors.

The chief remaining disagreement between the authors is the standard training set methodology (discussed in PCR with Residual Analysis in Practice subsection). In its current design, the use of PCR to analyze in vivo voltammetry data results in a trade-off between two separate guidelines for PCR: (1) matching instrumental and environmental conditions when generating calibration models and (2) independence between training set standards and data. Phillips and colleagues value the use of a training set that is generated from an independent source to that from which experimental data is collected. However, Wightman and colleagues maintain that the use of training sets obtained under unrepresentative conditions prevents definitive statements regarding statistical validation of PCR models when analyzing FSCV data, and has practical implications for signal extraction. Notably, this is true for training sets generated in vitro, in which it can be difficult to simulate the chemical environment of in vivo measurements, which is of particular importance for generating pH standards.

While the robustness of detection of striatal dopamine by FSCV in awake animals should inspire confidence, some of the greatest promise is beyond dopamine in the striatum. For detection of other electroactive neurochemicals in other brain regions,^155–158^ sensitivity and selectivity are more serious concerns because of lower analyte concentrations and a greater number of possible interferents. With this in mind, we believe that many of the caveats we have described in this Review will pose much greater challenges for these new applications. Specifically, key hardware changes could include more widespread use of chronically implanted electrode arrays that have moveable probes, and the use of stable polymer coatings on Ag/AgCl reference electrodes.
